# CMV Preventive and Treatment Studies in Children Undergoing Haematopoietic Stem Cell Transplant: A Systematic Review

**DOI:** 10.1002/rmv.70120

**Published:** 2026-02-18

**Authors:** Lara Fusani, Eleonora Fusco, Mariana Fonseca, Judith Breuer, Kanchan Rao, Luisa Galli, Alasdair Bamford, Seilesh Kadambari

**Affiliations:** ^1^ Infectious Diseases Unit Meyer Children's Hospital ‐ IRCCS Florence Italy; ^2^ School of Pediatrics University of Florence Florence Italy; ^3^ Department of Paediatric Infectious Diseases Great Ormond Street Hospital for Children NHS Foundation Trust London UK; ^4^ Department of Infection Immunity and Inflammation Great Ormond Street Institute of Child Health UCL London UK; ^5^ Department of Blood and Marrow Transplantation Great Ormond Street Hospital for Children NHS Foundation Trust London UK; ^6^ Department of Health Sciences University of Florence Florence Italy; ^7^ UCL Great Ormond Street Institute of Child Health London UK

**Keywords:** children, CMV, prevention, transplant, treatment

## Abstract

Cytomegalovirus (CMV) is one of the most important infectious causes of morbidity and mortality in children undergoing haematopoietic stem cell transplant (HSCT). However, the evidence for preventing and treating CMV disease in children in this context remains limited. We sought to review the current available evidence regarding the prevention and management of CMV infection and disease in children with HSCT and propose future research areas which could better inform evidence‐based management.

**Abbreviations**
AMCLI: Associazione Microbiologi Clinici Italiani
BMT: bone marrow transplant
CDV: cidofovir
CMV: cytomegalovirus
CMV‐CTL: cytotoxic T lymphocytes
CMV‐Ig: cytomegalovirus‐immunoglobulin
EBMT: European Society for Blood and Marrow Transplantation
ESCMID: European Society of Clinical Microbiology and Infectious Diseases
GCV: ganciclovir
GIMTO: Gruppo Italiano Trapianto di Midollo Osseo
GVHD: graft versus host disease
HSCT: haematopoietic stem cell transplant
IVIG: Intravenous immunoglobulins
LMV: letermovir
PCR: polymerase chain reaction
PRISMA: Preferred Reporting Items for Systematic Reviews and Meta‐Analyses
SITO: Società Italiana di Trapianto d’Organo
U‐DLI: unmanipulated donor lymphocyte
VGCV: valganciclovir
VSTs: virus‐specific T‐cells

## 1 Introduction

Cytomegalovirus (CMV) infection or reactivation is a significant cause of morbidity and mortality in children following allogeneic haematopoietic stem cell transplant (HSCT). Observational data in children after HSCT demonstrate that 20% of deaths with a known infectious cause are attributable to CMV [1, 2]. While some patients with CMV reactivation remain asymptomatic, others may develop symptomatic disease affecting any organ including colitis, hepatitis, encephalitis, retinitis and pneumonia [3, 4]. Other indirect effects associated with CMV reactivation may include an increased susceptibility to bacterial and fungal infections and/or graft versus host disease (GVHD) [5, 6]. Both CMV viraemia and disease have also been associated with increased length of hospital stay and additional cost [5, 6].

The risk of post‐transplant CMV reactivation and mortality is primarily driven by recipient CMV seropositivity, with the highest risk observed in CMV‐seropositive recipients transplanted from seronegative donors (D−/R+), while no increased risk has been consistently demonstrated in CMV‐seronegative recipients, irrespective of donor serostatus. [8].

Prophylaxis entails giving an antiviral medication for a predetermined amount of time with the goal of preventing CMV viraemia and can be distinguished between primary and secondary prophylaxis.

Primary prophylaxis typically consists of administering antiviral therapy from day 0 to approximately day +100 and is often pursued in patients at higher risk, including those with recent primary CMV infection immediately prior to transplant, recipients exposed to T‐cell depleting therapies, and recipients of T‐cell depleted, HLA‐mismatched, haploidentical or umbilical cord blood allografts [5, 6]. Clinical trials of antiviral prophylaxis have largely involved predominantly CMV‐seropositive recipients and/or donor‐seropositive transplants [5, 6]. Surveillance after prophylaxis can also be used in patients at risk for developing CMV viraemia, such as those with a high‐risk CMV profile. Secondary prophylaxis is defined as the prevention of recurrent disease following primary infection.

An alternative preventive strategy adopts a pre‐emptive approach. Pre‐emptive therapy includes monitoring for CMV viraemia at routine times post‐transplant and initiating antiviral therapy if viraemia is found at a predetermined threshold [5, 6]. This strategy minimises exposure to antiviral side effects and reduces the risk of antiviral resistance. Current ECIL‐10 guidelines recommend that CMV pre‐emptive therapy should be individualised, taking into account the quantitative nucleic acid testing (QNAT) platform and specimen matrix used (plasma vs. whole blood), the patient's risk of CMV disease, the presence or absence of antiviral prophylaxis, and clinically meaningful changes in CMV DNAemia over time, rather than applying a universal viral load threshold [5, 6]. Other guidelines have suggested a cut‐off between 500 and 1000 copies/ml, but initiating therapy when less than 1000 copies/ml may be associated with shorter duration [5, 6].

Despite CMV prevention strategies, CMV viraemia and disease continue to occur. Occasionally, CMV viraemia does not respond to first line antiviral agents either due drug resistant or refractory disease.

Refractory CMV infection is defined as CMV viraemia (DNAemia or antigenemia) that either increases (i.e., > 1 log10 increase in CMV DNA levels in the same blood compartment, measured using the same laboratory method or commercial assay) or persists (i.e., ≤ 1 log10 increase or decrease in CMV DNA levels) after at least 2 weeks of appropriately dosed antiviral therapy [5, 6].

In children undergoing HSCT, there is no consensus on prevention and treatment strategies with current available antiviral drugs. Furthermore, trials of newer antivirals such as letermovir or maribavir have been conducted mainly in adults [14, 15]. The aim of this systematic review is to assess the current evidence for prophylaxis, pre‐emptive therapy in CMV infection and treatment of established CMV disease post HSCT in children; highlighting research gaps that could be addressed to improve the evidence based management of CMV in this context.

## 2 Methods

### 2.1 Eligibility Criteria

This systematic review was performed according to the Preferred Reporting Items for Systematic Reviews and Meta‐Analyses (PRISMA)^16^ recommendations.

Inclusion criteria were: (i) study designs that included retrospective observational cohorts, prospective observational cohorts, case series and clinical trials in English and (ii) in children (0 – 18 years old) undergoing HSCT with (iii) clinical or virological assessment for CMV infection/disease. Other studies including case reports, conference abstracts, editorials and reviews and those in non‐humans were excluded.

### 2.2 Information Sources and Search Strategy

**FIGURE 1:**
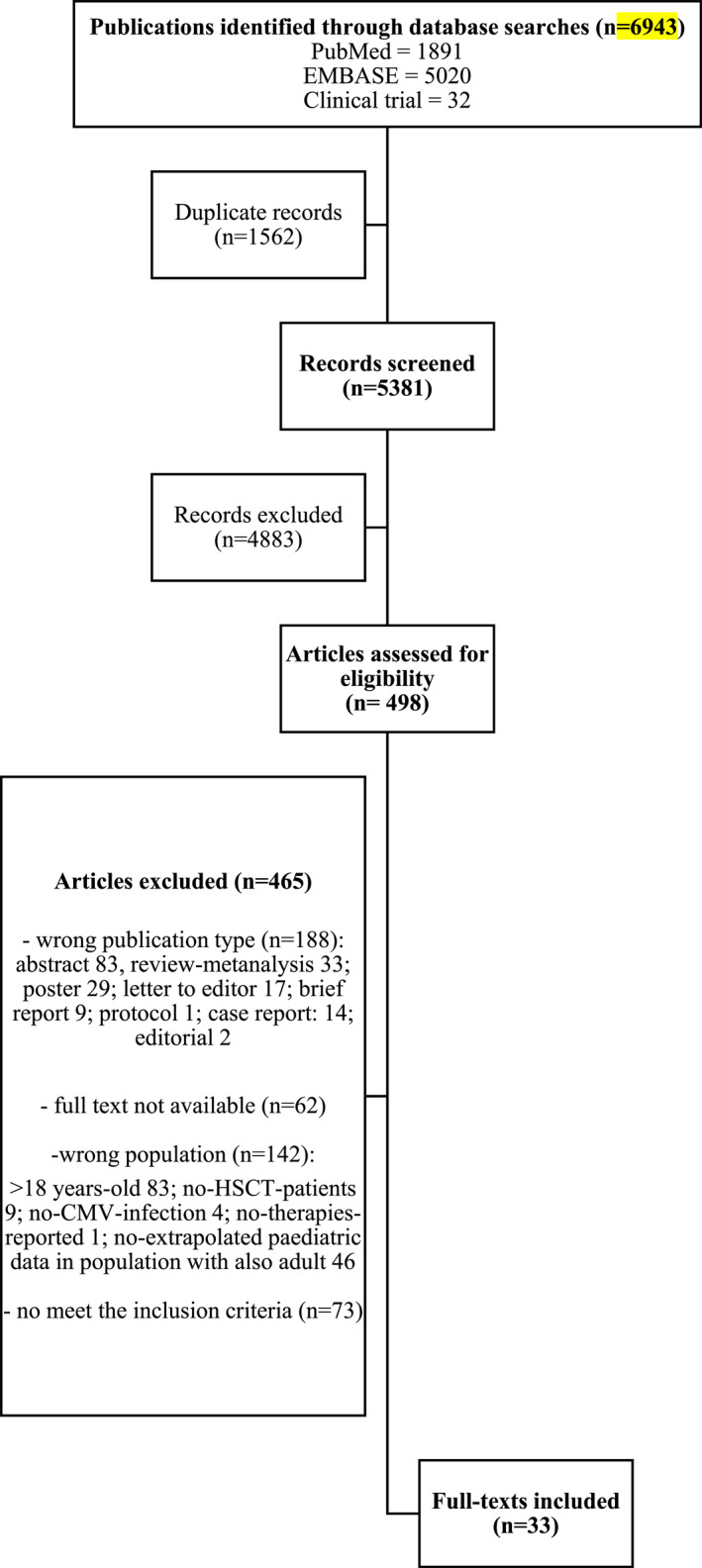
PRISMA flow chart of study screening and selection.

Studies in EMBASE, PubMed Medline and ClinicalTrials.gov were interrogated from inception up to the 31^st^ January 2025 using specific query strings (Figure 1). The following search terms were used: cytomegalovirus, CMV, bone marrow transplant, haematopoietic stem cell transplant, prevention, treatment, children and paediatric.

### 2.3 Selection and Data Collection Process

The title, abstract and full‐text screening were performed independently by three reviewers (L.F., E.F., M.F.) and use of an adjudicator (S.K.), according to the inclusion criteria, when needed. The full text review was conducted by S.K In case of disagreement between the reviewers.

Studies (e.g., retrospective observational cohorts, prospective observational cohorts, case series and clinical trials) in English and in children (0 – 18 years old) undergoing HSCT with clinical or virological assessment for CMV infection/disease were included. Single case reports, conference abstracts, editorials and narrative reviews, as well as non‐human studies, were excluded.

### 2.4 Data Items

Study characteristics (inclusion criteria, study design), studied population (sample size, age, sex) characteristics of CMV therapy (dose, treatment, duration), primary outcome, clinical and virological outcome, adverse events, and follow‐up were extracted from all studies included in this systematic review and reported in the data extraction table (Table 1).

**TABLE 1:** Summary of the CMV preventive and treatment studies in children undergoing HSCT, by chronological order.

Authors, country (year, ref.)	Study design and follow‐up	Population	CMV therapy	Primary outcome	Clinical outcome
Gudnason T. et al., Minnesota, USA (1989); [49]	Retrospective cohort study *36 months*	6 with CMV disease (total 12)	GCV (treatment)	Describe GCV therapy in a cohort of immunocompromised children	−2/6 clinically improved −4/6 died due to other underlying conditions
Maltezou HC, et al., Texas, USA (2000); [36]	Retrospective cohort study *24 months*	95 HSCT recipients	GCV (prophylaxis) Acyclovir (prophylaxis)	Describes incidence, clinical presentation, risk factors and outcome of viral infections in paediatric HSCT recipients	−70/95 viral infections −24/70 CMV infections −39/95 developed a viral disease −5/39 had CMV disease (4/5 through day 100 from transplant) –The highest case fatality was CMV related
Hazar V. et al., Turkey (2004); [27]	Retrospective cohort study *20 months* [2, 3, 4, 5, 6, 7, 8, 9, 10, 11, 12, 13, 14, 15, 16, 17, 18, 19, 20, 21, 22, 23, 24, 25, 26, 27, 28, 29, 30, 31, 32, 33, 34, 35, 36, 37, 38, 39, 40, 41, 42, 43, 44, 45, 46, 47, 48, 49, 50, 51, 52, 53]	76 HSCT	GCV High‐dose acyclovir	Evaluated high‐dose acyclovir and pre‐emptive GCV in the prevention of CMV disease in paediatric patients underwent peripheral blood stem cell transplantation	No CMV disease observed in the study period
Cesaro S., et al. Italy (2005); [38]	Retrospective cohort study *NR follow‐up*	12/30 CMV reactivation	CDV (preemptive) 10/12 received CDV: 9/10 s/third line therapy; 1/10 first line therapy	Evaluate safety and efficacy of CDV used pre‐emptively for CMV reactivation in children after HSCT	−5/8 complete response to CDV −3/8 switched to ganciclovir/foscarnet
Zaucha‐Prazmo A., et al. Poland (2005); [33]	Retrospective cohort study *9.5 months* [1, 2, 3, 4, 5, 6, 7, 8, 9, 10, 11, 12, 13, 14, 15, 16, 17, 18, 19, 20, 21, 22, 23, 24, 25, 26, 27, 28, 29, 30, 31, 32, 33, 34, 35, 36]	16 CMV reactivation (total patients:110)	Acyclovir (prophylaxis) GCV + IVIG (preemptive) Foscarnet (preemptive) 16 (reactivation): GCV + IVIG; 2/16 also foscarnet for resistance to GCV	Frequency and risk factors of CMV infection in children undergoing HSCT	4/16 died (no correlation with CMV)
Patel S.R et al., United Kingdom (2005); [29]	Retrospective cohort study *NR follow‐up*	108 patients: −41/108 CMV seropositive before transplant (donator or recipient) −67/108 CMV seronegative	Acyclovir and IVIG (prophylaxis) GCV (preemptive) Foscarnet (preemptive) –All population: prophylaxis −12/17 GCV −317 foscarnet −2/17 no treated	– Describe risk factors for CMV reactivation and for the development of CMV disease –Evaluate the efficacy of preemptive treatment	−2/17 developed CMV disease (one pneumonia and one retinitis) – no relationship found between the incidence of relapse and the timing of the first reactivation or duration of treatment
Giebel S. et al., Italy (2005); [34]	Prospective cohort study *NR follow‐up*	19/49 HSCT recipients with positive CMV pp65‐antigenemia	GCV (preemptive)	Evaluate prospectively if asymptomatic CMV infection in HSCT patients, treated pre emptively with GCV, influences T‐cell function	–No CMV disease developed in the cohort –Median time occurrence of CMV infection post‐ transplant was 37 days (13–65)
Behrendt C. E. et al., California, USA (2008); [37]	Retrospective cohort study *NR follow‐up*	140 HSCT recipients	GCV (preemptive) VGCV (preemptive) Foscarnet (preemptive)	Evaluate if CMV serostatus of donor and/or recipient affects the outcome of allogeneic HSCT in a cohort of children who underwent HSCT with routine use of CMV‐preemptive therapy	18/140 early CMV infection: −6/18 developed CMV disease (2 of them: died due to CMV) Observed a reduction in relapse with no increase in NRM (non‐relapse mortality) when the recipient and/or donor was CMV‐seropositive before transplantation
Yoon HS, et al. Korea (2008); [30]	Retrospective cohort study *34 months (range 1.9‐123 months)*	28 with CMV antigenemia detected at a median of 38 days (19–123 days) after HSCT; total patients 117	GCV (preemptive) Foscarnet (preemptive)	Analysed the frequency of CMV antigenemia among paediatric HSCT patients and identify risk factors for CMV infection	−4/28 died due to other underlying conditions −7/117 developed CMV disease at a median of 97 days post HSCT (34–120 days) –Three‐years survival rate was similar between CMV infection group (67%) and CMV non‐infection group (70%) after HSCT
Feuchtinger T. et al. Germany (2010); [48]	Multicenter retrospective cohort study *6 months*	8/18 (children/adult)	pp65‐specific T‐cells (treatment)	Describe adoptive T‐cell transfer as salvage treatment in SCT patients with chemo‐refractory CMV disease/infection	−7/8 positive response −1/8 no response (died due to sepsis) −1/8 died due to CMV disease
Tan P. L et al., Singapore (2014); [28]	Retrospective cohort study *26 months*	16/33 CMV DNAemia 1/16 CMV retinitis	Acyclovir + IVIG (prophylaxis) GCV (preemptive) VGCV (preemptive) –All population: prophylaxis –VGCV: 11/16 –GCV: 1/16 –VGCV and GCV: 4/16	Describe the prevalence of CMV infections in a cohort of paediatric HSCT patients	Patients treated with VGCV responded well to the treatment
Tavil B. et al., Turkey (2014); [32]	Retrospective cohort study *NR follow‐up*	66 HSCT	Acyclovir (prophylactic) GCV (preemptive)	Evaluate the effectiveness of acyclovir prophylaxis in the first 100 days after transplant and preemptive GCV for the prevention of CMV disease paediatric HSCT	−2/19 received foscarnet for 14 days for GCV resistant CMV antigenemia and/or disease with resolution of CMV antigenemia and/or disease –no death due to CMV reactivation and/or disease reported
Atay D. et al., Turkey (2015); [39]	Retrospective cohort study *NR follow‐up*	46/121 HSCT children with CMV reactivation Group 1: 22 (treated with GCV) Group 2: 24 (VGCV + GCV)	VGCV (preemptive) GCV (preemptive)	Describe one centre experiences of preemptive therapy with VGC + GCV in paediatric HSCT	– Median duration of therapy similar between the 2 groups: 24 days (12–69 days) in group II versus. 21 days (14–42 days) in group I **(*p* = 0.087)** **−**10/46 developed CMV disease (2 from Group 2 and 8 from Group 1)
Rowe R. G. et al., Massachusetts, USA (2016); [31]	Retrospective cohort study *NR follow‐up*	26 CMV infection (total 91 patients)	Acyclovir (prophylaxis) CMV hyperimmune globulin (preemptive/treatment) GCV (preemptive/treatment) Foscarnet (preemptive/treatment)	Identified risk factors for post‐SCT CMV infection that can be used to guide future CMV prophylaxis and treatment algorithms	–No effect of age in increased risk of CMV infection **(*p* = 0.40)** −1/26 died due to CMV pneumonia −1/26 resistant to GCV −1/26 developed CMV colitis
Avery, R. K. et al., Maryland, USA (2016); [42]	Retrospective cohort study *NR follow‐up*	4 with refractory CMV (total 17 HSCT patients)	Foscarnet (treatment)	Describe outcomes of HSCT recipients treated with foscarnet for treatment of resistant or refractory CMV	2/4 died
Ju H. Y. et al., Korea (2016); [41]	Retrospective cohort study *NR follow‐up*	87 with CMV antigenemia (total 130)	Acyclovir (prophylaxis) GCV (preemptive)	Evaluate efficacy and safety of half‐dose GCV prescribed preemptively after allo‐HSCT in children	−74/87 half‐dose GCV −10/87 GCV conventional dose No significative differences, matching of donor/recipient CMV serostatus (**p = 0.357**)
Foster J. H. et al., Texas, USA (2018); [43]	Retrospective cohort study *NR follow‐up*	150 HSCT 50 Group 1 100 Group 2	IVIG	Compare viral infection rate between prescribing IVIG trough IgG levels (Group 2) versus routine monthly administration (Group 1)	No significative differences detected in CMV infection rates (*p* = 0.43)
Uygun, V. et al., Turkey (2020); [45]	Case series *NR follow‐up*	5 CMV reactivation in HSCT recipients	UDL‐I treatment)	Describe the outcome of HSCT paediatric patients managed with UDL‐I	−4/5 survived without developing GVHD for a 3‐months period post‐U‐DLI −1/5 died due to pulmonary failure 7 days after UDL‐I
Hayes M. et al., Pennsylvania, USA (2021); [17]	Retrospective cohort study *180 days*	345 HSCT (244 with complete pharmaceutical data)	Acyclovir (prophylaxis) Valacyclovir (prophylaxis) VGCV (prophylaxis and preemptive) GCV (preemptive and treatment) Foscarnet (prophylaxis, preemptive and treatment) CDV (preemptive) −58/244 received specific CMV prophylaxis −54/244 received preemptive therapy	–Described rates and outcomes of CMV infection and disease in a paediatric cohort after HSCT –Reported adverse events associated antiviral therapies	– CMV disease developed in 10/89 (11.2%) with DNAemia −3/53 progressed to CMV disease during preemptive therapy
Styczyński J. et al., Poland, Italy, France, Switzerland, Sweden, Finland, Netherlands, Spain, United Kingdom (2021); [20]	Retrospective multicentre cohort study *NR follow‐up*	5 HSCT recipient (in total 49 with also adult)	LMV (primary or secondary prophylaxis) −2/5 (R+/D+) primary prophylaxis −3/5 secondary prophylaxis	Evaluated efficacy and safety of the use of LMV in off‐label indications	−3/5 alive, 2/5 died due to other underlying conditions
Ruan Y, et al., China (2022); [46]	Retrospective cohort study *60 months*	124/382 children with CMV infection	CMV‐CTL (treatment)	–Assess incidence and risk factors of CMV infection after allo‐HSCT –Evaluate safety and efficacy of donor‐derived and third‐party CMV‐CTL	−24/124 developed CMV disease −29/124 received CMV‐CTL – Therapy effective in 26/29 patients – No GVHD progress registered after CMV‐CTL infusion −6/29 died (3 due to CMV progress)
Körholz K. F. et al., Germany (2022); [18]	Retrospective cohort study *12 months*	17 HSCT patients	LMV (prophylaxis/preemptive) −5/17 received prophylactic LMV −12/17 received preemptive LMV	Evaluate effectiveness, safety and tolerance of prophylactic and pre‐emptive off‐label use of LMV in paediatric allogeneic HCT patients	−1/12 changed to ganciclovir due to absent response −1/12 LMV discontinue due to disease relapse −14/17 completed therapy with LMV
Łojko A. et al. Poland (2022); [19]	Retrospective cohort study *8 months (1‐24 months)*	7 HSCT patients (46 total with adults)	LMV (prophylaxis)	Describe effectiveness and safety of LMV prophylaxis	No patients died due to CMV disease
Heston S. M et al. North Carolina, USA (2022); [40]	Retrospective cohort study *24 months*	244/969 developed CMV viraemia 25 (12–40) days after HSCT	GCV (treatment/preemptive) Foscarnet (treatment/preemptive) CMV Ig (treatment/preemptive) VGCV (treatment/preemptive) −77/244 GCV −14/244 foscarnet −89/244 GCV and foscarnet −4/244 CMV Ig −3/244 VGCV −1/244 VGCV and CMV immune globulin −56/244 no directed treatment for CMV viraemia	Compared the incidence of adverse events among HSTC children with CMV viraemia treated with GCV or foscarnet	GCV treatment had longer duration than foscarnet (median [IQR], 39.5 [17–64] vs 22 [11–40.5] days) **(*p* < 0.0001)**
Szmit Z. et al., Poland (2022); [35]	Retrospective cohort study *36 months*	27 CMV reactivation (total patients 94)	Acyclovir (prophylaxis) GCV (preemptive) Foscarnet (preemptive, second line agent; treatment) CMV IgG hyperimmunoglobulin (preemptive second line, treatment)	Evaluate the incidence and outcome of CMV reactivation in non‐malignant paediatric HSCT	11/27 preemptive GCV insufficient: −6/11foscarnet −5/11 foscarnet + CMV IgG hyperimmunoglobulin
Jiang W. et al., Australia (2022); [47]	Clinical trial *12 months*	3 CMV reactivation (total patients 30)	VSTs (treatment)	Assessed safety and efficacy of third‐party VSTs administered with antivirals in HSCT patients	All 3 patients sustained complete response
Geurten C. et al., Birmingham (United Kingdom) (2022); [44]	Retrospective cohort study *NR follow‐up*	76 HSCT children 49/76 at risk for CMV reactivation	CMV‐Ig (prophylaxis) 39/49 do not received CMV‐Ig (CMV‐Ig ‐) 10/49 started CMV‐Ig (CMV‐Ig +)	Evaluate benefit of CMV‐Ig prophylaxis on incidence and severity of CMV reactivation.	−4/76 patients developed CMV disease −2/4 died due to CMV – none of the CMV‐Ig + group developed CMV disease or died −4/39 from the CMV‐Ig ‐ developed CMV disease and 5/39 died – Time to recovery shorter in CMV‐Ig + **(*p =* 0.31)**
Daukshus N. P. et al., New York (USA) (2022); [22]	Retrospective case series	10 HSCT adolescents	LMV (prophylaxis)	Describe a single centre experience with LMV prophylaxis	–LMV started at a median of 19 days after HSCT (7–49 days) –In the cohort none developed CMV disease −2/10 required CMV treatment after LMV discontinuation
Kuhn A. et al., Minnesota (USA) (2023); [21]	Retrospective case series *NR follow‐up*	9 HSCT children	LMV (primary or secondary prophylaxis) −2/9 started LMV as primary prophylaxis due to intolerance to VGCV −2/9 received LMV as secondary prophylaxis −5/9 started LMV as primary prophylaxis	Describe a single centre experience with LMV for CMV prophylaxis in children underwent HSCT	‐no CMV disease developed in the cohort
Galaverna F. et al., Italy (2024); [23]	Retrospective cohort study *24 months*	87 HSCT children	LMV (primary prophylaxis): 39/87 cases LMV (secondary prophylaxis): 26/87 cases LMV (pre‐emptive): 18/87 cases LMV treatment 4/87 cases	Assess the efficacy and safety profile of prophylactic and therapeutic off‐label use of letermovir in paediatric allogenic HCT patients.	–no discontinuation due to toxicity –none of the patients had CMV symptomatic reactivaction during LMV primary prophylaxis −10 patient developed asymptomatic CMV reactivation −1 patient developed CMV primary infection after drug discontinuation
Groll A. et al., multicenter (2024); [24]	Phase 2b, open label, single arm CT study *Up to 48 weeks post transplant*	28 HCT adolescents	LMV (prophylaxis)	Evaluate PK, safety and efficacy of letermovir in participants from birth < 18 yo who were at risk of developing CMV infection and/or disease following allogenic HCT	–Administration of adult letermovir doses in adolescent cohort result in exposures within adult clinical programme margins –Safety and efficacy similar to adults
Pfeiffer T. et al., MO (USA) (2024); [25]	Retrospective cohort study *up to day +100 post transplant*	22 HCT children	LMV primary prophylaxis	Assess safety and efficacy of LTM as CMV prophylaxis in children following allo‐HCT	−9/22 developed CMV reactivation, from which 5/9 had low viral load and 4/9 progressive viraemia –No patients developed CMV associated end organ disease
Wang Q. et al., Sozhou (China) (2025); [26]	Retrospective case‐control study *12 months*	178 HCT children	LMV prophylaxis 80/178 received LMV prophylaxis 98/178 control, no prophylaxis	Evaluate the safety and efficacy of LMV prophylaxis in paediatric patients	–The cumulative incidence of CMV reactivation was significantly lower in the letermovir group compared to the control group (27.8% vs. 60.2%, *p* < 0.001) – no patients developed CMV disease – no serious adverse events reported

Abbreviations: BID: twice a day; CDV: cidofovir; CMV: cytomegalovirus; CMV‐CTL: CMV‐specific cytotoxic T lymphocytes; CMV‐Ig: CMV‐Hyperimmunoglobulin; GCV: ganciclovir; Ig: immunoglobulins; IV: intravenous; IVIG: Immunoglobulins intravenous; LMV: Letermovir; NR: not reported; PO: oral; QID: four times a day; SCT: halogenic stem cell transplantation; TID: three times a day; UDL‐I: unmanipulated donor lymphocyte; VGCV: valganciclovir; VSTs: Adoptive T‐cell therapy with virus‐specific T‐cells (VSTs).

## 3 Results

### 3.1 Study Selection

Our initial search identified 6943 publications, of which 33 (0.48%) met the criteria for inclusion (Figure 1). Based on title/abstract screening and duplicate records (1562 papers), 6445 studies (92.8%) were excluded. 498 papers were assessed for eligibility, and 465 of them were excluded at the full‐text review stage (93.4%).

Overall, 29 retrospective cohort studies, 1 case series, 1 prospective cohort study and 2 clinical trials were included. Studies were conducted between 1/1/1989 and 31/01/2025. While most studies lasted for 12 or 24 months, the longest study duration reached 60 months. Studies were mostly conducted in the USA (*N* = 11). Details of the included studies and a summary of the characteristics of each selected study are shown in Table 1.

### 3.2 Prophylaxis

#### 3.2.1 Ganciclovir (GCV)

We only found one study where ganciclovir (GCV) was used in a CMV‐specific prophylaxis regimen (acyclovir/valacyclovir or valganciclovir to day 7 followed by foscarnet to engraftment followed by valganciclovir/ganciclovir until day +100) and prescribed in 23.8% (58/244) patients at high risk for CMV infection [17].

#### 3.2.2 Letermovir (LMV)

Letermovir (LMV) was used in nine studies [18, 19, 20, 21, 22, 23, 24, 25, 26].

In recent years, there has been an increase in the number of published studies looking at letermovir prophylaxis in children undergoing allogenic HSCT. Overall, no children receiving LMV prophylaxis developed CMV‐associated end‐organ disease. CMV reactivation varied between studies and was described in larger cohort studies.

Of note, from the larger cohort studies, Galaverna et al. [23], LMV was used as primary and secondary prophylaxis in 39 and 26 cases, respectively. Median duration was 100 days (14–256) for primary and 96 days (8–271) for secondary prophylaxis. None of the patients experienced clinically significant CMV reactivation during LMV primary prophylaxis; one patients developed breakthrough viraemia during secondary prophylaxis and 10 experienced asymptomatic CMV reactivation.

Wang et al. [26] retrospectively analysed a cohort with 178 children who underwent allo‐HSCT; 80 patients received prophylactic LMV while 98 patient without prophylaxis served as the control group. The cumulative incidence of CMV viral reactivation was significantly lower in the letermovir group compared to the control group (27.8% vs. 60.2% group, *P* < 0.001) and no patients developed CMV disease. The median time to CMV reactivation was later in the letermovir group (139 days, range 88–301) than in the control group (34 days, range 7–153).

Additionally, across the studies, LMV was described to be well tolerated and no serious nor significant adverse events were reported. Further details as describe in Table 2.

**TABLE 2:** CMV prophylaxis characteristics of CMV studies in children undergoing HSCT, by chronological order.

Authors, country (year, ref.)	Study design and follow‐up	CMV therapy	Dose and duration	Virological outcome	Adverse effects
Maltezou H. C. et al., Texas, USA (2000); [36]	Retrospective cohort study *24 months*	GCV (prophylaxis) Acyclovir (prophylaxis)	5 mg/kg/day 3 times(until October,1992) or 5 times (from November,1992) per week until +100 days 500 mg/m^2^ iv tid until + 30 days	NR	NR
Hazar V. et al., Turkey (2004); [27]	Retrospective cohort study *20 months* [2, 3, 4, 5, 6, 7, 8, 9, 10, 11, 12, 13, 14, 15, 16, 17, 18, 19, 20, 21, 22, 23, 24, 25, 26, 27, 28, 29, 30, 31, 32, 33, 34, 35, 36, 37, 38, 39, 40, 41, 42, 43, 44, 45, 46, 47, 48, 49, 50, 51, 52, 53]	High‐dose acyclovir (prophylaxis)	10 mg/kg IV tid or 20 mg/kg PO qid, oral prophylaxis continued until 6 months post‐transplant	‐ Rate of CMV activation undergoing high‐dose acyclovir prophylaxis: 19.7% (15/76), with median onset of 22 days after transplant	NR
Patel S. R. et al., United Kingdom (2005); [29]	Retrospective cohort study *NR follow‐up*	Acyclovir and IVIG (prophylaxis) –All population: prophylaxis	CMV +: 500 mg/m^2^ tid, 3 months; CMV – :10 mg tid, 3 months	−17/41 (41.46%) developed CMV reactivation (mean time: 44 +/− 31.6 days after transplant)	NR
Zaucha‐Prazmo A. et al. Poland (2005); [33]	Retrospective cohort study *9.5 months* [1, 2, 3, 4, 5, 6, 7, 8, 9, 10, 11, 12, 13, 14, 15, 16, 17, 18, 19, 20, 21, 22, 23, 24, 25, 26, 27, 28, 29, 30, 31, 32, 33, 34, 35, 36]	Acyclovir (prophylaxis)	NR	−16/110 reactivation under prophylaxis (13.63%)	NR
Tan P. L. et al., Singapore (2014); [28]	Retrospective cohort study *26 months*	Acyclovir + IVIG (prophylaxis) – All population: prophylaxis	NR	7/16 (43.75%) had > 1 episode of CMV DNAemia	NR
Tavil B. et al., Turkey (2014); [32]	Retrospective cohort study *NR follow‐up*	Acyclovir (prophylaxis)	250 mg/m^2^/dose iv tid (from conditioning regimen to neutrophil engraftment) or oral (after neutrophil engraftment); until +100 days (40–50 mg/kg/day, oral)	NR	NR
Ju H. Y. et al., Korea (2016); [41]	Retrospective cohort study *NR follow‐up*	Acyclovir (prophylaxis)	500 mg/m^2^ IV every 8 h, until day 90 post‐transplant	−51/74 (68.92%) cleared CMV	NR
Rowe R. G. et al., Massachusetts, USA (2016); [31]	Retrospective cohort study *NR follow‐up*	Acyclovir (prophylaxis)	500 mg/m^2^ iv tid, until + 30 days	‐CMV infection developed at a median of 46 days after graft infusion (9–127 days)	NR
Foster J. H. et al., Texas, USA (2018); [43]	Retrospective cohort study *NR follow‐up*	IVIG (prophylaxis)	Group 2: 500 mg/kg to maintain IgG > 400 mg/dL until day + 90	– CMV reactivation in Group 1: 30% – CMV reactivation in Group 2: 24%	NR
Hayes M. et al., Pennsylvania, USA (2021); [17]	Retrospective cohort study *180 days*	Acyclovir (prophylaxis) Valacyclovir (prophylaxis) VGCV (prophylaxis) Foscarnet (prophylaxis) −58/244 received specific CMV prophylaxis	Oral: < 20 kg: 100 mg bid; 20–40 kg: 200 mg bid; ≥ 40 kg: 400 mg bid; IV: 250 mg/m^2^/dose tid NR 1 month–16‐year‐old: once daily dose (mg) = 7 x body surface area x creatinine clearance (maximum dose: 900 mg/day); adults: 900 mg once daily 90 mg/kg/dose daily	–CMV DNAemia detected in 89/345 (25.8%) –CMV infection rate was high for those receiving a CMV specific prophylaxis regimen (50%)	NR
Styczyński J. et al., Poland, Italy, France, Switzerland, Sweden, Finland, Netherlands, Spain, United Kingdom (2021); [20]	Retrospective multicentre cohort study *NR follow‐up*	LMV (primary or secondary prophylaxis) −2/5 (R+/D+) primary prophylaxis −3/5 secondary prophylaxis	1 Patient 480 mg/day, 2 patients 240 mg/day, 2 patients 120 mg/day; orally	no breakout of CMV infection on children received LMV as primary prophylaxis	2/5 (40%) nausea or vomit
Daukshus N. P. et al., New York (USA) (2022); [22]	Retrospective case series	LMV (prophylaxis)	480 mg/kg/day; PO/IV 240 mg/kg/day when used with cyclosporine.	−1/10 (10%) had intermittent, low level CMV DNAemia	−1/10 (10%) vomiting and severe nausea
Geurten C. et al., Birmingham (United Kingdom) (2022); [44]	Retrospective cohort study *NR follow‐up*	CMV‐Ig (prophylaxis) 39/49 do not received CMV‐Ig (CMV‐Ig ‐) 10/49 started CMV‐Ig (CMV‐Ig +)	0.5 g/kg (1 mL/kg) fortnightly for 6 doses	−20/76 (26.32%) reactivation of CMV −3/10 (30%) reactivation of CMV in CMV‐Ig + versus. 15/39 in CMV‐Ig – (*p* = 0.62) – CMV reactivation developed earlier in CMV‐Ig + (*p =* 0.68)	‐no adverse effects due to CMV‐Ig infusion reported
Körholz K. F. et al., Germany (2022); [18]	Retrospective cohort study *12 months*	LMV (prophylaxis/preemptive)	> 30 kg: 480 mg (adult dose) PO < 30 kg and > 18 kg: 240 mg PO < 18 kg: 120 mg PO IV similar doses (except for < 12‐year‐old and > 30 kg: 120 mg)	−1/5 (20%) transient increases in CMV load –‐10/12 (83.33%) no detectable CMV at the end of the treatment – At the end of the prophylactic treatment none had a detectable CMV load	No patients developed LMV adverse events
Łojko A. et al. Poland (2022); [19]	Retrospective cohort study *8 months (1‐24 months)*	LMV (prophylaxis)	4 patients: 240 mg/day; 2 patients: 120 mg/day; 1 patient: 60 mg/day median duration of LMV treatment 90 days (range 6–270 days)	NR	No patients developed AEs due to LMV
Szmit Z. et al., Poland (2022); [35]	Retrospective cohort study *36 months*	Acyclovir (prophylaxis)	10–15 mg/kg/day starting from day 10 of transplant	NR	NR
Kuhn A. et al., Minnesota (USA) (2023); [21]	Retrospective case series *NR follow‐up*	LMV (primary or secondary prophylaxis) −2/9 started LMV as primary prophylaxis due to intolerance to VGCV −2/9 received LMV as secondary prophylaxis −5/9 started LMV as primary prophylaxis	Children weighing < 30 kg 240 mg/kg/day; PO Children weighing ≥ 30 kg 480 mg/kg/day; PO	−1/9 (11.11%) reactivation of CMV	−2/9 (22.22%) mild, transient, spontaneously solved transaminitis
Galaverna F. et al., Italy (2024); [23]	Retrospective cohort study *24 months*	LMV (primary prophylaxis): 39/87 cases LMV (secondary prophylaxis): 26/87 cases	Data on LMV daily dosage available for 47/87 cases: ≥ 30 kg: 480 mg (adult dose) – 13 cases 18 ‐30 Kg: 50% reduced adult dose – 27 cases < 18 Kg: 25% reduced adult dose – 6 cases 1 case aged 8 months and 6 kg received 80 mg Median duration 100 days for LMV primary prophylaxis and 96 days for LMV secondary prophylaxis	– No CMV clinical symptomatic reactivation during LMV primary prophylaxis – After discontinuation of LMV primary prophylaxis 4/39 (10.26%) developed asymptomatic CMV reactivation −1/26 (3.85%) developed breakthrough infection during secondary prophylaxis – After discontinuation of LMV secondary prophylaxis 6/26 (23.08%) developed asymptomatic CMV reactivation	No discontinuation due to severe toxicity was reported
Groll A. et al., multicenter (2024); [24]	Phase 2b, open label, single arm CT study *Up to 48 weeks post transplant*	LMV prophyalxis: 28 adolescents	Body weight 28.7 Kg – 95 Kg): 480 mg (without CsA) Or 240 mg (with CsA)	NR	No new major safety concerns were reported, similarly to profile seen in adult participants. No evidence of myelotoxicity, hepatotoxicity or nephrotoxicity.
Pfeiffer T. et al., MO (USA) (2024); [25]	Retrospective cohort study	LMV prophylaxis 22 children	≥ 12 yo: 480 mg daily 2‐11 yo; ≥ 18 to < 30 kg: 240 mg daily 2‐11 yo; 11 to < 18 kg: 120 mg daily LMV was started at a median of 2 days prior to stem cell infusion, range −101 and + 38	−7/22 (31.82%) never developed CMV viraemia −9/22 (40.91%) developed CMV reactivation, from which 5/9 had low viral load and 4/9 progressive viraemia –No patients developed CMV associated end organ disease	LMV was well tolerated and without reported adverse events
Wang Q. et al., Sozhou (China) (2025); [26]	Retrospective case‐control study *12 months*	LMV prophylaxis 80/178 received LMV prophylaxis 98/178 control, no prophylaxis	≥ 50 kg: 480 mg daily ≥ 30 to < 50 kg: 240 mg daily 10 to < 30 kg: 240 mg every other day < 10 kg: 80 mg daily If the GVHD prophylaxis medication contains cyclosporine, the dose of LMV should be halved LMV was continued until 100 days post‐transplant	–The cumulative incidence of CMV reactivation was significantly lower in the letermovir group compared to the control group (27.8% vs. 60.2%, *P* < 0.001) – median time to CMV reactivation was later in LMV group (139 days, range 88 – 301) than in the control group (34 days, range 7–153) – there was a trend towards a loer peak CMV viral load in LMV group (*P* < 0.001) – no patients developed CMV disease	– no interruption in treatment due to adverse events due to LMV prophylaxis – No serioud adverse events reported

Abbreviations: BID: twice a day; CDV: cidofovir; CMV: cytomegalovirus; CMV‐CTL: CMV‐specific cytotoxic T lymphocytes; CMV‐Ig: CMV‐Hyperimmunoglobulin; GCV: ganciclovir; Ig: immunoglobulins; IV: intravenous; IVIG: Immunoglobulins intravenous; LMV: Letermovir; NR: not reported; PO: oral; QID: four times a day; SCT: halogenic stem cell transplantation; TID: three times a day; UDL‐I: unmanipulated donor lymphocyte; VGCV: valganciclovir; VSTs: Adoptive T‐cell therapy with virus‐specific T‐cells (VSTs).

#### 3.2.3 Acyclovir

Acyclovir has been well studied as a prophylactic agent in children post‐HSCT patients. In a cohort of 110 children, monitored twice a week through CMV antigenemia, prophylaxis with high dose of acyclovir (10 mg/kg IV tid or 20 mg/kg PO qid) was administrated. In 14.55% (16/110), viral reactivation was seen [27]. Prophylaxis with high dose acyclovir was evaluated in another cohort of 76 children: CMV reactivation was monitored with weekly antigenemia, and the rate of reactivation was similar to the previous study (19.7%, 15/76); no CMV disease was observed during the follow‐up [27].

Another study, using acyclovir with Intravenous immunoglobulins (IVIG) in patients at risk for CMV reactivation was associated with a higher rate of CMV reactivation (16/33, 48.5%) [28]. In this study, CMV viraemia was monitored weekly through PCR.

Patel et al., retrospectively enroled 108 participants and divided them in two sub‐cohorts according to CMV serology: 38% (41/108) were CMV seropositive before HSCT and 62% (67/108) CMV seronegative. High dose acyclovir and IVIG were prescribed to the group with positive CMV serology, while the negative group received standard dose of acyclovir and IVIG. CMV reactivation was detected through weekly antigenemia. The reactivation rate was 41.5% (17/41) in the CMV serology positive group, while no reactivations were observed in the negative cohort [29].

Acyclovir prophylaxis was also prescribed in a Korean cohort of 130 HSCT children, with a reactivation of CMV antigenemia in 67% (87/130); prophylaxis was administered from day 0 to +90 days [30]. In three other studies, acyclovir was prescribed as prophylaxis [31, 32, 33]. One study monitored CMV reactivation through antigenemia assays [34], the other two used serial PCR [31, 35]. All three studies had significant differences in duration of prophylaxis but the rate of CMV reactivation was similar: 28.8% (19/66) [34], 28.7% (27/94) [35], 28.6% (26/91) [31], respectively.

Maltezou et al., described a prophylactic regimen that combined GCV and acyclovir for CMV‐seropositive HSCT patients, or for those with a CMV‐seropositive donor. Participants received GCV from admission to 2 days before transplant, then acyclovir until engraftment. GCV was then reintroduced until 100 days post‐transplant. With this regimen, 34% (24/70) developed CMV infection and 13.8% CMV disease [36].

A more recent study compared different prophylaxis regimens according to the risk of CMV infection. In this study, 68.0% (166/244) received standard dose of acyclovir/valacyclovir, 23.8% (58/244) received CMV‐specific prophylaxis (valganciclovir/GCV) and 8.2% (20/244) did not receive prophylaxis. CMV viraemia rates were the similar in those who did not receive any prophylaxis (4/20, 20.0%) and acyclovir (33/166, 19.9%), while in the CMV‐specific prophylaxis group viraemia reached a rate of 50% (29/58)^17^.

Among the selected studies, 798 patients received prophylaxis with acyclovir: 567 acyclovir monotherapy (186 patients high‐dose), 90 GCV and acyclovir (acyclovir administered until engraftment), 141 patients acyclovir and IVIG; overall, 63.3% (505/798) of patients did not show any CMV reactivation.

### 3.3 Pre‐Emptive Therapy

#### 3.3.1 Ganciclovir

Ganciclovir (GCV) is the most studied pre‐emptive agent [17, 27, 28, 29, 30, 31, 32, 33, 34, 35, 37, 38, 39, 40, 41]. In one retrospective cohort study, GCV was preemptively prescribed with IVIG in 14.5% (16/110) participants with CMV viral reactivation while on acyclovir prophylaxis. Overall, 25% (4/16) showed a recurrence of CMV viraemia after the end of the preemptive treatment and one case developed antiviral resistance [27].

One study used preemptive GCV in 12/17 patients. Overall, 5/17 had a CMV recurrence after a mean of 20 +/− 61 days from the end of preemptive therapy (with GCV or foscarnet). Among them, 3/5 were successfully treated with another course of GCV and 2/5 developed CMV disease (one pneumonia and the other one retinitis) [32].

One study aimed to evaluate the efficacy and safety of preemptive treatment with half dose of GCV (5 mg/kg once daily, 6 days/week). Among 87 children with CMV reactivation, 74/87 received half‐dose of GCV and 10/87 the standard dose (5 mg/kg twice daily, 7 days/week). In the half‐dose group, 51/74 patients cleared CMV viraemia, while in 23/74 patients the dose was increased to full dose, due to increases in CMV levels. No significant association was found between either dosing group and time to clear virus or CMV recurrence (*p =* 0.831) [40].

Another study reported excellent outcomes in participants receiving GCV preemptive treatment. All 15/76 patients with CMV antigenemia were treated with a 14‐day course of GCV, cleared CMV and no recurrence was described [17].

Hayes et al. [17], in a retrospective cohort study of 54/244 children who had preemptive treatment, included 18/54 who received at least a course of GCV (5 mg/kg/dose twice daily for 14–21 days): 1/18 received only GCV (no CMV reactivation detected), 6/18 received GCV followed by valganciclovir (VGCV) and foscarnet (4/6 CMV reactivation), 1/18 received GCV and then VGCV, cidofovir and foscarnet and then developed CMV viraemia. In total, 8/18 were treated with a combination of GCV and VGCV (no reactivation in 5/8) and 2/18 GCV combined with foscarnet (1/2 CMV reactivation). Side effects were reported in only 2/18 patients receiving GCV: one patient showed renal dysfunction and GCV was first reduced then stopped, the other one had neutropenia and the drug was stopped. There was no difference in CMV‐reactivation between participants who had preemptive therapy and those that did not (21/53 vs. 4/14, *p* = 0.544) [17].

Atay et al. [39], published a retrospective cohort study of preemptive therapy in 46/121 children with CMV reactivation. 22/46 (Group 1) were treated with GCV and 24/46 (Group 2) received GCV then VGCV. The median treatment duration was similar in group I [respectively 21 days (14–42 days) and group II [24 days (12–69 days, (*p* = 0.087)], and no myelotoxicity was reported in either group (*p* > 0.05). No significant differences were seen between groups with respect to CMV viraemia (*p* = 0.71), or CMV recurrence (6/22, 4/24; *p* = 0.3). Overall, 10/46 children (8 from the first group and 2 from the second one) developed CMV disease.

GCV was successfully administered in a cohort of 19/49 HSCT paediatric patients in a prospective observational cohort study [34]. CMV antigenemia was detected at a median of 37 days (13–65 days) after transplant. All patients had viral resolution after 17 days (5–40 days) and none developed CMV disease. Another study showed a high rate of CMV viraemia clearance within 14 days of transplant in 15/20 (75%) preemptively treated with GCV and CMV hyperimmunoglobulin; the median CMV viraemia clearance time was around 9.5 days (1–34 days) [40].

Less encouraging data were reported in a retrospective cohort study: preemptive GCV was administered to 27/94 children after CMV reactivation, which occurred 27.5 days post‐HSCT (24–78 days); However, 11/27 developed GCV resistance; 1/27 developed CMV pneumonia [35].

Behrendt et al., described 140 children receiving preemptive therapy with GCV or VGCV. The cumulative incidence of disease/viraemia 100 days after the transplant was 12.9% (+/−3.0%), and 18/140 developed early infection [median 42 (15–77) days] [37].

In summary, GCV was used as the only preemptive agent in 96 patients with an overall clinical response in 70/96 (72.9%). The most commonly used dose for GCV across all studies was 5 mg/kg/day (Table 3).

**TABLE 3:** CMV Preemptive strategies of CMV studies in children undergoing HSCT.

Authors, country (year, ref.)	Study design and follow‐up	Preemptive monitoring/threshold	CMV therapy	Dose and duration	Virological outcome	Adverse effects
Behrendt C. E. et al., California, USA (2000); [37]	Retrospective cohort study *NR follow‐up*	Preemptive monitoring: CMV PCR Threshold: 2 consecutive positive PCR tests, or 1 PCR with viral load ≥ 5000/mL or ≥ 1000/mL during high dose corticosteroid therapy	GCV (preemptive) VGCV (preemptive) Foscarnet (preemptive)	5 mg/kg bid, for 7 days; then 5 weeks of maintenance with GCV given once daily, 5 days per week 450 mg/m^2^ bid for 7 days, then 5 weeks of maintenance with VGCV once daily, 5 days per week NR	–Incidence of CMV viraemia/disease 12.9 (± 3.0) % + 100 days –Time to onset of early CMV infection median 42 days (15–77)	NR
Hazar V. et al., Turkey (2004); [27]	Retrospective cohort study *20 months* [2, 3, 4, 5, 6, 7, 8, 9, 10, 11, 12, 13, 14, 15, 16, 17, 18, 19, 20, 21, 22, 23, 24, 25, 26, 27, 28, 29, 30, 31, 32, 33, 34, 35, 36, 37, 38, 39, 40, 41, 42, 43, 44, 45, 46, 47, 48, 49, 50, 51, 52, 53]	Preemptive monitoring: pp65 antigenemia assay. (If WCC was not sufficiently high for the pp65 assay, CMV PCR was used) Threshold: 10 or more positive cells per 200,000 was defined as high‐risk and treated.	GCV (preemptive)	5 mg/kg bid for 14 days followed by 5 mg/kg/day until negative CMV antigenemia	–CMV antigenemia resolved in all patients treated with pre‐emptive GCV –None of the children developed rising antigenemia during GCV therapy –No recurrence of antigenemia after GCV treatment	NR
Cesaro S., et al. Italy (2005); [38]	Retrospective cohort study *NR follow‐up*	Preemptive monitoring: pp65 antigenemia assay. Threshold NR	CDV (preemptive) 10/12 received CDV: 9/10 s/third line therapy; 1/10 first line therapy	Induction:5 mg/kg once weekly x2; Maintenance: 2 to 4 fortnightly doses at 3–5 mg/kg *median number of doses per course: 5 (range 1‐6)*	8/10 positive CMV antigenemia assay when CDV was started	NR
Zaucha‐Prazmo A., et al. Poland (2005); [33]	Retrospective cohort study *9.5 months* [1, 2, 3, 4, 5, 6, 7, 8, 9, 10, 11, 12, 13, 14, 15, 16, 17, 18, 19, 20, 21, 22, 23, 24, 25, 26, 27, 28, 29, 30, 31, 32, 33, 34, 35, 36]	Preemptive monitoring: pp 65 antigenemia assay Threshold: At least 2 infected cells out of 4 × 10^5^ leucocytes	GCV + IVIG (preemptive) Foscarnet (preemptive) 16 (reactivation): GCV + IVIG; 2/16 also foscarnet for resistance to GCV	NR	−4/16 (25%) recurrence of pp65 antigenemia after preemptive therapy	NR
Patel S.R et al., United Kingdom (2005); [29]	Retrospective cohort study *NR follow‐up*	Preemptive monitoring: pp65 antigenemia assay. Threshold NR	GCV (preemptive) Foscarnet (preemptive) −12/17 GCV −317 foscarnet −2/17 no treated	5 mg/kg bid 60 mg/kg tid *First CMV reactivation treated for 17.7 +/− 9 days*	−17/41 developed CMV reactivation (mean time: 44 +/− 31.6 days after transplant) −67/108 CMV seronegative: no reactivation −5/17 relapsed after preemptive treatment 20 +/− 61 days after the first reactivation	1/17 (5.9%) switched GCV to foscarnet due to myelosuppression
Giebel S. et al., Italy (2005); [34]	Prospective cohort study *NR follow‐up*	Preemptive monitoring: pp65‐antigenemia assay Threshold: When two or more pp65‐positive PBL were detected, or when detection of one pp65‐positive PBL was confirmed on the following 2–3 days by the same or a greater number of pp65‐positive PBL.	GCV (preemptive)	5 mg/kg bid until pp65‐antigenemia clearance	–GCV was effective in all cases with CMV antigenemia clearance –median time from the first positive test to the first of two negative antigenemia tests was 17 days [5, 6, 7, 8, 9, 10, 11, 12, 13, 14, 15, 16, 17, 18, 19, 20, 21, 22, 23, 24, 25, 26, 27, 28, 29, 30, 31, 32, 33, 34, 35, 36, 37, 38, 39, 40]	NR
Yoon H. S. et al. Korea (2008); [30]	Retrospective cohort study *34 months (range 1.9‐123 months)*	Preemptive monitoring: pp65 antigenemia assay Threshold: ≥ 2 positive cells per 200,000 PMN cells detected. If antigenemia of only one positive cell per 200,000 PMN cells detected, retest after 2 days and considered positive if second test was above one positive cell.	GCV (preemptive) Foscarnet (preemptive)	Induction: 5 mg/kg bid for 7–14 days; if antigenemia decreased or negative→ maintenance: 5 mg/kg/day for 7 days; If antigenemia positive after 7–14 days → continued to complete a 21‐day course or until negative NR *Median antiviral treatment duration was 26 days (7–162 days)*	24/28 cleared CMV with antiviral treatment	3/28 (10.7%) switched ganciclovir to foscarnet due to bone marrow suppression
Tan P. L. et al., Singapore (2014); [28]	Retrospective cohort study *26 months*	Preemptive monitoring: CMV PCR Threshold: Detection of any DNAemia	GCV (preemptive) VGCV (preemptive) –VGCV: 11/16 –GCV: 1/16 –VGCV and GCV: 4/16	NR	7/16 had > 1 episode of CMV DNAemia	NR
Tavil B. et al., Turkey (2014); [32]	Retrospective cohort study *NR follow‐up*	Preemptive monitoring: CMV PCR Threshold: ≥ 400 copies/mL on 2 tests or ≥ 1000 copies/mL on 1 test	GCV (preemptive) Foscarnet (preemptive)	5 mg/kg bid for 14 days For 14 days; dose NR	−19/66 had CMV DNAemia median follow‐up 381 days (100–720 days) –CMV reactivation detected after a median 5 weeks (2–9 weeks) after HSCT –GCV administered for a median of 14 days [4, 5, 6, 7, 8, 9, 10, 11, 12, 13, 14, 15, 16, 17, 18, 19, 20, 21] until the DNAemia resolved	NR
Atay D. et al., Turkey (2015); [39]	Retrospective cohort study *NR follow‐up*	Preemptive monitoring: CMV PCR Threshold > 1000 DNA copies/mL	VGCV (preemptive) GCV (preemptive)	15–18 mg/kg bid 5 mg/kg bid	–No significative difference between Group 1 and 2 in CMV DNA load **(*p* = 0.71)** –Median time to the first negative CMV DNA: 14 days (I) and 12 days (II) –No significative difference about the incidence of a second CMV reactivation **(*p* = 0.3)**	–No myelotoxicity observed during both treatment **(*p* > 0.05)** –No patients developed adverse events in both groups
Ju H. Y. et al., Korea (2016); [41]	Retrospective cohort study *NR follow‐up*	Preemptive monitoring: pp65 antigenemia assay. (CMV PCR done if antigenemia positive) Threshold: Lower limit of CMV PCR detection 100 copies/mL	GCV (preemptive)	5 mg/kg once daily, 6 days/week Conventional dose: 5 mg/kg every 12 h, 7 days/week	−51/74 cleared CMV −23/74 advanced to full GCV dose due to the increase of CMV antigenemia levels	Neutropenia: 70.6% in group of conventional GCV doses and in 51.0% in the group of half doses (***p* = 0.065**)
Rowe R. G. et al., Massachusetts, USA (2016); [31]	Retrospective cohort study *NR follow‐up*	Preemptive monitoring: CMV PCR Threshold > 500 copies/mL	CMV hyperimmune globulin (preemptive/treatment) GCV (preemptive/treatment) Foscarnet (preemptive/treatment) VGCV (preemptive)	100 mg/kg 3 times per week for 14 days 5 mg/kg bid, minimum 14 days 60 mg/kg tid iv, minimum 14 days 500 mg/m^2^ daily *All 26 patients received a 14‐day course of antiviral + thrice weekly immune globulin therapy*	–CMV infection developed at a median of 46 days after graft infusion (9–127 days) −20/26 cleared CMV after 14 days −6/26 did not clear CMV within 14 days −7/26 recurrence at a median of 33 days (9–74 days)	NR
Hayes M. et al., Pennsylvania, USA (2021); [17]	Retrospective cohort study *180 days*	Preemptive monitoring: CMV PCR Threshold: Any qualitative detection of CMV by PCR	VGCV (preemptive) GCV (preemptive) Foscarnet (preemptive) CDV (preemptive) −54/244 received preemptive therapy	Adults: 900 mg twice daily for minimum of 14–21 days > 3 months: 5 mg/kg/dose bid for 14–21 days 60 mg/kg/dose tid OR 90 mg/kg/dose bid for 14–21 days 5 mg/kg IV weekly for 2 doses, then every other week plus probenecid	‐CMV DNAemia detected in 89/345 (25.8%)	NR
Körholz K. F. et al., Germany (2022); [18]	Retrospective cohort study *12 months*	Preemptive monitoring: CMV PCR Threshold NR	LMV (prophylaxis/preemptive) −5/17 received prophylactic LMV −12/17 received preemptive LMV	> 30 kg: 480 (adult dose) PO < 30 kg and > 18 kg: 240 mg PO < 18 kg: 120 mg PO IV similar doses (except for < 12‐year‐old and > 30 kg: 120 mg)	−1/5 transient increases in CMV load –10/12 no detectable CMV at the end of the treatment –At the end of the prophylactic treatment none had a detectable CMV load −3/14 transient reactivations after the end of treatment (no need for adjunctive treatment)	NR
Heston S. M. et al. North Carolina, USA (2022); [40]	Retrospective cohort study *24 months*	Preemptive monitoring: CMV PCR Threshold: 2 consecutive weeks of CMV PCR > 500 IU/mL or after 1 measurement > 1000 IU/mL	GCV (treatment/preemptive) Foscarnet (treatment/preemptive) CMV Ig (treatment/preemptive) VGCV (treatment/preemptive) −77/244 GCV −14/244 foscarnet −89/244 GCV and foscarnet −4/244 CMV Ig −3/244 VGCV −1/244 VGCV and CMV immune globulin −56/244 no directed treatment for CMV viraemia	NR	NR	–GCV compared with foscarnet, was associated with lower Incidence rates of thrombocytopaenia ([IRR], 0.38; 95% CI, 0.15–0.97), electrolyte AEs (IRR, 0.42; 95% CI, 0.24–0.75), endocrine AEs (IRR, 0.52; 95% CI, 0.34–0.79), and renal AEs (IRR, 0.36; 95% CI, 0.19–0.65) –No differences in the incidence rates of leucopenia, anaemia, and gastrointestinal observed between foscarnet and GCV
Szmit Z. et al., Poland (2022); [35]	Retrospective cohort study *36 months*	Preemptive monitoring: CMV PCR Threshold > 500 copies/mL	GCV (preemptive) Foscarnet (preemptive, second line agent; treatment) CMV IgG hyperimmunoglobulin (preemptive second line, treatment)	5 mg/kg bid 180 mg/kg/daily NR		NR
Galaverna F. et al., Italy (2024); [23]	Retrospective cohort study *24 months*	Preemptive monitoring: CMV PCR Threshold, reference used by centre ‐ reported median CMV viraemia of 2070 (range 1430‐83083 copies IU/mL)	LMV preemptive −18/87 cases	≥ 30 kg: 480 mg (adult dose) < 30 kg: 10 mg/Kg/day Started at median interval from HCT of 48 days and median duration of treatment 66 days	−17/18 cleared CMV viraemia −1/18 developed CMV symptomatic disease	No discontinuation due to severe toxicity was reported

Abbreviations: BID: twice a day; CDV: cidofovir; CMV: cytomegalovirus; CMV‐CTL: CMV‐specific cytotoxic T lymphocytes; CMV‐Ig: CMV‐Hyperimmunoglobulin; GCV: ganciclovir; Ig: immunoglobulins; IV: intravenous; IVIG: Immunoglobulins intravenous; LMV: Letermovir; NR: not reported; PO: oral; QID: four times a day; SCT: halogenic stem cell transplantation; TID: three times a day; UDL‐I: unmanipulated donor lymphocyte; VGCV: valganciclovir; VSTs: Adoptive T‐cell therapy with virus‐specific T‐cells (VSTs).

#### 3.3.2 Valganciclovir (VGCV)

We found Valganciclovir (VGCV) was used as preemptive therapy in six studies [16, 18, 20, 30, 31, 39], including four studies which evaluated VGCV in combination with other antiviral therapies [16, 20, 30, 39].

In one study, VGCV was given to 30/54 children who received at least one course of preemptive therapy [17]. VGCV was the only preemptive drug in 4/30 children (no CMV reactivation). Overall, 5/30 treated developed side effects: 1/5 neutropenia and 4/5 renal dysfunction; VGCV was stopped in the patient with neutropenia and in 1/4 with renal dysfunction, while in the others the dose was reduced. None of these patients progressed to CMV disease.

In summary, 74 patients received VGCV. The cohort analysed by Behrendt et al. was not included as the author did not provide a breakdown of the number of patients receiving VGCV and GCV [37]. Only 5 children (from cohorts of two papers [17, 31]) received VGCV alone, with an overall response of 100% (clinical for the first cohort [17] and virological^31^ for the second one). Most patients (36 from different cohorts [17, 28, 39] as shown in the paragraph of GCV) received both VGCV and GCV, without reactivation in 84.4% (27/32; for patients from one paper [39] the specific response to VGCV was not available). VGCV dosing was varied across the studies (900 mg two times daily for 14–21 days, or 15–18 mg/kg two times daily, or 500 mg/m^2^ daily for 14 days, or 450 mg/m^2^ two times daily for 7 days and then 5 days/week for 5 weeks, Table 3).

#### 3.3.3 Foscarnet

We identified nine studies which investigated the preemptive use of foscarnet [16, 20, 22, 27, 31, 32, 34, 35].

In a cohort of 16 children with CMV reactivation, foscarnet was administered in two patients after resistance to GCV was detected. No data were reported on the duration of pre‐emptive treatment [27].

Patel et al. described the administration of foscarnet in three children [29]. One child received 4 weeks of IV foscarnet for CMV retinitis which developed during a second reactivation with complete resolution. A second patient was treated with foscarnet due to significant myelosuppression due to his disease and a third was switched from GCV after developing associated marrow toxicity. No further disease after therapy was reported in any of the three cases.

In another retrospective cohort study, foscarnet was given as second‐line treatment in 2/19 patients with CMV reactivation for ganciclovir‐resistant CMV antigenemia and/or disease with complete response [34].

Szmit et al., administered foscarnet as second line in 11/27 patients (6/11 received only foscarnet and 5/11 also CMV Ig) due to GCV resistance; no data were reported about its efficacy [35].

Hayes et al., published a retrospective observational study in a cohort of 345 children undergoing HSCT (244 with complete pharmaceutical data), to evaluate outcomes of CMV infection and disease along with adverse effect related to antiviral treatment [17]. Overall, CMV viraemia was detected in 25.8% (89/345 children), among them 10/89 developed CMV disease (11.2%). Foscarnet was prescribed as single preemptive agent for 24 patients, while 20 children received both foscarnet and ganciclovir/valganciclovir. Notably, among all the 54 children preemptively treated, 3 patients treated with only foscarnet, progressed to CMV disease. In addition, the authors showed that 15 patients, who have received foscarnet developed reactivation during follow‐up (5 patients received only foscarnet, the remainder other drugs with foscarnet as first‐line in 8/10 patients). Only one patient received foscarnet as second‐line without reactivation detected. The overall rate response was 59.1% (26/44).

Heston et al., enroled a large cohort of 969 HSCT children to evaluate to evaluate risk factors for CMV viraemia and treatment‐associated adverse events in patients receiving ganciclovir or foscarnet for CMV [18]. Among them, 244 developed CMV viraemia and 180/244 received antiviral treatment: 14/180 were treated with foscarnet, 4/180 with CMV immune globulin, 3/180 with VGCV, 1/180 with VGCV in combination with CMV immune globulin, 77/180 with GCV and 89/180 with GCV and foscarnet. No information was available on the comparative antiviral efficacy. However, the duration of ganciclovir treatment was significantly longer than foscarnet (median [IQR], 39.5 [17–64] vs. 22 [11–40.5] days) (*p* < 0.0001)). Moreover, while foscarnet use was associated with higher incidence of thrombocytopaenia (incidence rate ratio [IRR], 0.38; 95% CI, 0.15–0.97), renal (IRR, 0.36; 95% CI, 0.19–0.65), endocrine (IRR, 0.52; 95% CI, 0.34–0.79) and electrolyte (IRR, 0.42; 95% CI, 0.24–0.75) abnormalities, no significative differences were observed with respect to leucopenia or anaemia, suggesting comparable bone marrow toxicity profiles. No differences in gastrointestinal adverse events were detected either.

Rowe et al., identified 26/91 HSCT children with CMV infection. Five patients received foscarnet as first line drug and CMV hyperimmune globulin; 80% (4/5) had negative viraemia within 14 days; another patient received foscarnet as second‐line drug (after GCV) and cleared CMV [31].

In a Korean paediatric cohort of 117 patients post HSCT, 28 developed CMV antigenemia: preemptive treatment with GCV or foscarnet was tolerated without adverse events, and treatment was administered for a median of 26 days (range: 7–162 days); Foscarnet was prescribed as second‐line due to bone marrow suppression caused by GCV in 3/28 children. No data were available about the efficacy of foscarnet or GCV, but overall 85.7% (24/28) of patients treated with antivirals cleared CMV, and 7/117 developed CMV disease 97 days post HSCT (34–120 days) [22].

In summary, foscarnet alone as first‐line preemptive agent was administrated in 41 patients [16, 18, 20, 32] with 69.23% (18/26) showing a good response (2/2 [32], 16/24 [17]) and 15/41 outcomes not available [18, 20]. Second‐line preemptive therapy was prescribed in four children (from data available [16, 27, 39]) with a good virological response in all. The dosing of foscarnet varied between studies from 60 to 180 mg/kg as shown in Table 3.

#### 3.3.4 Cidofovir (CDV)

Cesaro et al. evaluated the efficacy and safety profile of preemptive Cidofovir (CDV) in a cohort of 8 children (administering 5 mg/kg once weekly for 2 weeks and 2 to 4 fortnightly doses at 3–5 mg/kg, with a median number of doses per course of 5) as second‐ or third‐line therapy after being treated with FSC and/or GCV [42]. In this study, a complete response was defined as complete clearance of CMV antigenemia. The response rate was 62.5% (5/8), while 3/8 patients were switched successfully to ganciclovir or foscarnet due to persistent/increasing CMV antigenemia; no nephrotoxicity was reported in this group. In another study, CDV was used in one case as third‐line agent, after foscarnet and GCV, without adverse effects [17]. CMV was monitored by testing CMV viraemia using PCR and in both studies CDV was used at the same dose (Table 3).

#### 3.3.5 Intravenous Immunoglobulins (IVIG)

The role of IVIG in the management of CMV in HSCT patients has been investigated in four studies [29, 31, 33, 43] and discussed previously in this review. Only one study, published by Foster er al., used IVIG as CMV prophylaxis in paediatric HSCT patients, administering 500 mg/kg to maintain IgG > 400 mg/dL until day +90. In this retrospective cohort study, 150 children were recruited into two groups accordingly: Group 1 (50 patients) received a monthly routine infusion of IVIG, Group 2 (100 children) received IVIG based on specific IgG levels. No significant differences in CMV reactivation was detected in either group (CMV reactivation was 30% in Group 1% and 24% in Group 2, *p* = 0.43) [43].

#### 3.3.6 CMV‐Immunoglobulin (CMV‐Ig)

Three studies evaluated the role of CMV‐Ig role in management of disease and have been discussed previously [18, 31, 35]. The benefit of CMV‐Ig prophylaxis in the incidence and severity of CMV in HSCT children has been evaluated in a retrospective cohort of 76 children [44]. The authors identified 49 participants at risk for CMV reactivation. Overall, 10/49 received the CMV‐Ig infusion (Group 1), while 39/49 did not receive prophylaxis (Group 2). No side effects related to CMV‐Ig infusion were reported and no significant differences were found between either group in occurrence of CMV reactivation (respectively 3/10 in first group and 15/39 in the second one, *p =* 0.62). In Group 1, no cases developed CMV disease or died. In Group 2, 4/39 developed CMV disease and 5/39 died. Moreover, although not statistically significant, time to recovery was shorter in patients receiving CMV‐Ig [21 (± 7) days versus. 51.35 (± 54.97) days, *p =* 0.31). CMV hyperimmune globulin has also been used as adjunctive therapy in combination with antivirals, particularly in earlier retrospective cohorts [31].

#### 3.3.7 Other Anti‐Virals Used as Pre‐Emptive Therapy

Galaverna et al. [23] described 18 patients who received LMV as monotherapy, while 4 patients received foscarnet in combination with LMV treatment. Among pre‐emptive therapy group, LMV was started with a median CMV viraemia of 2070 (range 1430 – 83,083 copies IU/mL); median duration of treatment was 66 days. Seventeen out of 18 patients cleared CMV viraemia.

### 3.4 Treatment

#### 3.4.1 Foscarnet

Avery and colleagues described using foscarnet as treatment for refractory/resistant CMV disease in 4 children: CMV was cleared in 75% of children (3/4), but eventually 2/4 died [42].

#### 3.4.2 Adoptive Immunotherapy

Uygun et al., in a case series of five paediatric HSCT patients with CMV infection or disease analysed outcomes after the infusion of unmanipulated donor lymphocyte (U‐DLI). Overall, CMV viraemia significantly reduced after U‐DLI, 4/5 children survived without developing GVHD for a 3 month‐period and 1/5 died due to pulmonary failure 7 days after UDL‐I [45].

A study published by Ruan and colleagues evaluated the safety and efficacy of commercial CMV‐CTL (cytotoxic T lymphocytes) in a cohort of 124/382 children with CMV disease. In total, 29/124 (18/29 with CMV disease) received CMV‐CTL. The infusions were well tolerated, without any side effects or GVHD reported. The treatment was effective in 26/29 patients, but 3/6 died due to CMV disease [46]. Jiang et al., published a prospective clinical trial to evaluate efficacy and safety of third‐party virus‐specific T‐cells (VSTs) used in combination with standard antiviral treatment. Among a cohort of 30 HSCT patients, the authors identified three children with CMV reactivation. All cases showed a sustained and complete virological response and no adverse events related to the infusion were reported [47].

A multicenter retrospective study conducted in Germany in a cohort of 18 HSCT patients (8/18 children) described transfer of pp65‐specif T‐cells as treatment for chemo‐refractory CMV infection or disease. In the paediatric cohort, 4/8 cleared CMV infection, 3/8 showed a significant reduction of viraemia and one patient died of CMV disease [48].

#### 3.4.3 Other Antivirals Used as CMV Treatment (Ganciclovir, Valganciclovir, Letermovir)

With respect to other treatment studies, Tavil and colleagues administered GCV in 19/66 children undergoing HSCT who developed CMV reactivation after a median of 5 weeks (2–9 weeks) post‐HSCT [32]. GCV was prescribed for a median of 14 days (4–21 days) until viral resolution. Two cases developed resistance to GCV and were switched to foscarnet.

In another retrospective cohort study, conducted in a smaller cohort of 33 patients, with 4 cases receiving two HSCT, CMV viraemia was detected in 43% (16/33) patients and 1/16 developed CMV disease (retinitis). In total, 4/16 were treated with a combination of IV GCV and oral valganciclovir (VGCV), 11/16 with only VGCV and 1/16 with only IV GCV; CMV viraemia recurred in 7/16 (44%) children [39].

Early experience with GCV as treatment for CMV infection in paediatric HSCT recipients was reported by Gudnason et al. in a retrospective cohort study, showing virological response in five out of 6 treated patients [49].

Limited use of LMV as CMV treatment was reported by Galaverna et al. [23] and described in Table 4. We found no studies on the use of Maribavir in children with CMV disease.

**TABLE 4:** CMV treatment summary of CMV studies in children undergoing HSCT.

Authors, country (year, ref.)	Study design and follow‐up	CMV therapy	Dose and duration	Virological outcome	Adverse effects
Avery, R. K. et al., Maryland, USA (2016); [42]	Retrospective cohort study *NR follow‐up*	Foscarnet (treatment)	Dose NR; duration: median of 32 days (range: 6–193)	3/4 cleared CMV viraemia	
Szmit Z. et al., Poland (2022); [35]	Retrospective cohort study *36 months*	Foscarnet (preemptive, second line agent; treatment) CMV IgG hyperimmunoglobulin (preemptive second line, treatment)	180 mg/kg/daily NR		
Hayes M. et al., Pennsylvania, USA (2021); [17]	Retrospective cohort study *180 days*	GCV (treatment) Foscarnet (treatment)	> 3 months: 5 mg/kg/dose daily for 7 days/week OR 6 mg/kg/dose for 5 days/week 90–120 mg/kg/dose daily		Adversee events: 19.2%
Heston S. M. et al. North Carolina, USA (2022); [40]	Retrospective cohort study *24 months*	GCV (treatment/preemptive) Foscarnet (treatment/preemptive) CMV Ig (treatment/preemptive) VGCV (treatment/preemptive) −77/244 GCV −14/244 foscarnet −89/244 GCV and foscarnet −4/244 CMV Ig −3/244 VGCV −1/244 VGCV and CMV immune globulin −56/244 no directed treatment for CMV viraemia	NR		–GCV compared with foscarnet, was associated with lower Incidence rates of thrombocytopaenia ([IRR], 0.38; 95% CI, 0.15–0.97), electrolyte AEs (IRR, 0.42; 95% CI, 0.24–0.75), endocrine AEs (IRR, 0.52; 95% CI, 0.34–0.79), and renal AEs (IRR, 0.36; 95% CI, 0.19–0.65) –No differences in the incidence rates of leucopenia, anaemia, and gastrointestinal observed between foscarnet and GCV
Rowe R. G. et al., Massachusetts, USA (2016); [31]	Retrospective cohort study *NR follow‐up*	CMV hyperimmune globulin (preemptive/treatment) GCV (preemptive/treatment) Foscarnet (preemptive/treatment)	100 mg/kg 3 times per week for 14 days 5 mg/kg bid, minimum 14 days 60 mg/kg tid iv, minimum 14 days *All 26 patients received a 14‐day course of antiviral + thrice weekly immune globulin therapy*	–CMV infection developed at a median of 46 days after graft infusion (9–127 days) −20/26 cleared CMV after 14 days −6/26 did not clear CMV within 14 days −7/26 recurrence at a median of 33 days (9–74 days)	
Gudnason T. et al., Minnesota, USA (1989); [49]	Retrospective cohort study *36 months*	GCV (treatment)	7.5 mg/kg/die (range 2.5–15 mg/kg/day) in 2–3; duration 5–47 days	5/6 responded virologically to GCV	none developed absolute neutropenia
Galaverna F. et al., Italy (2024); [23]	Retrospective cohort study *24 months*	LMV (treatment) −4/87 cases	≥ 30 kg: 480 mg (adult dose) < 30 kg: 10 mg/Kg/day Started at median interval from HCT of 65 days and median duration of treatment 18 days	−2/4 cleared CMV viraemia −2/4 did not responde to the treatment	No discontinuation due to severe toxicity was reported
Feuchtinger T, et al. Germany (2010); [48]	Multicenter retrospective cohort study *6 months*	pp65‐specific T‐cells (treatment)	Mean dose 21 × 10^3^/kg; 1 infusion	−4/8 cleared CMV −3/8 significant reduction (> 1 log) of CMV load	
Ruan Y, et al., China (2022); [46]	Retrospective cohort study *60 months*	CMV‐CTL (treatment)	0.95 × 1^7^/kg	Median time of CMV viraemia occurrence: 48 days after allo‐HSCT	No adverse reaction found during the transfusion of CMV‐CTL
Uygun, V. et al., Turkey (2020); [45]	Case series *NR follow‐up*	UDL‐I (treatment)	5 × 10^4^/kg except for one patient who received 3 × 10^5^/kg	CMV viraemia dramatically reduced after UDL‐I	
Jiang W. et al., Australia (2022); [47]	Clinical trial *12 months*	VSTs (treatment)	Each dose: 2.0 × 10^7^/m^2^ partially HLA‐matched CMV specific VSTs		No adverse events attributed to VSTs infusion

Abbreviations: BID: twice a day; CDV: cidofovir; CMV: cytomegalovirus; CMV‐CTL: CMV‐specific cytotoxic T lymphocytes; CMV‐Ig: CMV‐Hyperimmunoglobulin; GCV: ganciclovir; Ig: immunoglobulins; IV: intravenous; IVIG: Immunoglobulins intravenous; LMV: Letermovir; NR: not reported; PO: oral; QID: four times a day; SCT: halogenic stem cell transplantation; TID: three times a day; UDL‐I: unmanipulated donor lymphocyte; VGCV: valganciclovir; VSTs: Adoptive T‐cell therapy with virus‐specific T‐cells (VSTs).

## 4 Discussion

To our knowledge, this is the first systematic review to comprehensively describe clinical outcomes in the prevention and treatment of CMV in children after HSCT. In our systematic review, we found monitoring of CMV for pre‐emptive therapy varied between the different studies. Different assays were used to assess CMV viraemia, which included pp65 antigenemia assay or CMV PCR assays. Furthermore, different thresholds were used to start pre‐emptive therapy and these also varied across studies. Ganciclovir/VGCV and foscarnet are the most commonly studied agents in pre‐emptive studies and acyclovir and LMV in prophylactic studies [12, 14]. Children appeared to tolerate pre‐emptive antiviral therapy with GCV, with few reported adverse effects and good reported outcomes in preventing CMV disease [16, 17, 19, 20, 32, 34, 39, 40]. GCV had a good safety profile, with neutropenia reported to be the most common side effect [16, 40]. Lack of response to GCV treatment occurred most commonly due to antiviral resistance.

We also found good clinical outcomes when GCV resistant cases were switched to foscarnet [27, 34]. Most studies used foscarnet as second‐line therapy, especially in patients with profound myelosuppression, pre‐engraftment and in whom GCV would not be tolerated, with CMV clearance and a low rate of progression to disease [22, 32, 34, 35]. We found foscarnet had a good safety profile, even when combined with other antivirals [16, 22]. Only one study compared adverse events between GCV and foscarnet in children with CMV viraemia and showed foscarnet was more commonly associated with thrombocytopaenia, renal and endocrine abnormalities, while no differences were seen in leucopenia, anaemia, and gastrointestinal side effects between the two drugs [18].

In the adult HSCT population, letermovir has emerged as the preferred prophylactic agent. In the paediatric setting, the evidence is limited. This review identified nine studies which reported good outcomes in children receiving LMV as a pre‐emptive and prophylactic strategy and protecting against development of CMV infection. LMV was well tolerated in all the studies that reported use in children [18, 19, 20, 21, 22] and is a promising candidate that should be studied in larger paediatric focused trials. Optimal letermovir dosing in young children in not established. Results of ongoing trials (NCT03940586, NCT05711667) evaluating the use of letermovir in children are awaited in order to confirm potential implemetnation in the prophylaxis setting. Furthermore, the optimal duration of prophylaxis has not been completely defined and an eventual extension of prophylaxis from 100 to 200 days is currently under evaluation in a clinical trial in order to reduce late‐onset CMV infection (NCT03930615).

ECIL‐10 recommends letermovir as the preferred strategy for primary CMV prophylaxis in CMV‐seropositive adult allo‐HCT recipients (grade A1 evidence) [9]. This guidance is based on a phase 3‐double blind‐trial in an adult population [14], where LMV reduced clinically proven infection versus placebo at 24 weeks, with no major toxic effects and with all‐cause mortality reduced at week‐48 after transplantation. In the trial, LMV was administered at a dose of 480 mg per day (or 240 in patients taking cyclosporine), starting a median of 9 days after HSCT and for 14 weeks. LMV resistance has been described, but is uncommon in the setting of prophylaxis strategies [46]. LMV has excellent oral bioavailability, rarely requires dose adjustments in the context of renal/hepatic impairment and has excellent efficacy and safety profiles from phase three trials and real world data [20]. The recent recommendations from the 10^th^ European Conference on Infections in Leukaemia^9^ provides some guidance for letermovir prophylaxis and also supports the notion that RCT data are required to help inform further practice in children. A survey of the Infectious Diseases Working Party of European Society for Blood and Marrow Transplantation (EBMT), highlighted its widespread use in large adult centres as a first line agent in prophylactic approaches^38^ which provides further encouragement to investigate LMV in younger age groups.

IVIG is widely used as a preventive agent, however we did not find any obvious benefit. The British haematology, BMT and virology guidelines also do not recommend the routine use of IVIG in preventing CMV viraemia (grade 1A evidence) [50]. CMV‐CTL's have been shown to have some efficacy in small studies to prevent disease and treating refractory infection. Further observational studies, across large international networks, could obtain useful data in informing wider use of CMV‐CTLs in hard‐to‐treat infections.

We only identified paediatric treatment trial evaluating GCV and foscarnet and no trial data on VGCV, CDV or maribavir. We found that studies used different durations of therapy, varied doses and trial endpoints were often inconsistent which makes comparison difficult.

VGCV is widely used as a suitable oral therapy in the treatment of mild‐moderate CMV disease [51]. ECIL‐10 guidelines highlight the importance of using VGCV, as an alternative to IV ganciclovir/foscarnet, in the absence of severe gastrointestinal GvHD (grade AII) [9].

Some centres use CDV, for instance during periods of myelosuppression [52]. For second‐/third‐line therapy, cidofovir and (in selected situations) ganciclovir plus foscarnet are options [9].

A phase III trial with the aim of determining the safety, tolerability, and pharmacokinetics of maribavir in children 0 to < 18 years of age (NCT05319353) has been planned (Takeda. A Phase 3, open‐label, single‐arm, repeated‐dose study to evaluate the safety and tolerability, pharmacokinetics, and antiviral activity of maribavir for the treatment of cytomegalovirus (CMV) infection in children and adolescents who have received a hematopoietic stem cell transplant (HSCT) or a solid organ transplant (SOT).

The British haematology, BMT and virology guidelines suggest using higher doses of GCV or foscarnet in combination with IVIG for primary CMV disease, while patients are treated pre‐emptively or with progressive disease and awaiting resistance testing [50]. Guidelines provide some support for using IVIG or CMV‐Ig only in the treatment of CMV pneumonia [9], while other paediatric guidelines on supportive therapy recommended IVIG therapy only in cases of hypogammaglobulinemia [51]. In our review, we found no trials evaluating the use of IVIG in the treatment of CMV infection or disease.

We used a broad search strategy and attempted to interrogate relevant studies in depth. This review has highlighted various limitations in our evidence base management: significant heterogeneity between studies, varied inclusion criteria, different definitions for infection/disease, different assays and thresholds for pre‐emptive treatment and doses/duration of treatment used. The significant differences between studies did not allow for comparison of outcomes or infer more effective preventive/treatment regimens. The availability of a consensus standard for molecular assays for CMV and viral load measurements would facilitate cross comparisons and facilitate future multicentre trials [53].

This review supports the rationale for a higher quality of evidence to be obtained from pragmatically done randomised controlled treatment trials. Determining the optimal duration of prophylaxis, antiviral dosing, and minimising the risk of adverse effects during treatment are all clinical challenges that could be addressed using large registry data. Future pharmacokinetic/pharmacodynamic and drug monitoring studies could also be done to better understand how letermovir might be introduced more widely in the paediatric setting.

Further studies are also necessary in order to better define the use of different antivirals in children, especially for the treatment of refractory or resistant disease. For instance, it would be useful to compare standard treatment (i.e., GCV) with newer antivirals like maribavir or combination regimens. Innovative trial methodology such as adaptive and pragmatic trials, and rigorous analysis of real‐world data should be prioritized given the inherent challenges of doing double blind randomized controlled trials in children undergoing HSCT.

## Author Contributions

**Lara Fusani:** methodology, writing and original draft. **Eleonora Fusco:** methodology, writing and original draft. **Mariana Fonseca:** methodology and writing. **Judith Breuer:** methodology, writing – reviewing and editing. **Kanchan Rao:** methodology, writing – reviewing and editing. **Luisa Galli:** methodology, writing – reviewing and editing. **Alasdair Bamford:** methodology, writing – reviewing and editing. **Seilesh Kadambari:** conceptualisation, methodology, writing, original draft.

## Data Availability

Data sharing not applicable to this article as no datasets were generated or analysed during the current study.
